# Sequential Fusion Filter for State Estimation of Nonlinear Multi-Sensor Systems with Cross-Correlated Noise and Packet Dropout Compensation

**DOI:** 10.3390/s23104687

**Published:** 2023-05-12

**Authors:** Liguo Tan, Yibo Wang, Changqing Hu, Xinbin Zhang, Liyi Li, Haoxiang Su

**Affiliations:** 1 Laboratory for Space Environment and Physical Sciences, Harbin Institute of Technology, Harbin 150001, China; tanliguo@mail.ru (L.T.); xinbinzhang@hit.edu.cn (X.Z.); 2 School of Astronautics, Harbin Institute of Technology, Harbin 150001, China; 22s104130@stu.hit.edu.cn; 3 School of Information Engineering, Southwest University of Science and Technology, Mianyang 621010, China; hu_changqing@mails.swust.edu.cn (C.H.); shx@mails.swust.edu.cn (H.S.)

**Keywords:** sequential fusion estimation, correlated noise, packet dropout compensation, multi-sensor systems, nonlinear filtering

## Abstract

This paper is concerned with the problem of state estimation for nonlinear multi-sensor systems with cross-correlated noise and packet loss compensation. In this case, the cross-correlated noise is modeled by the synchronous correlation of the observation noise of each sensor, and the observation noise of each sensor is correlated with the process noise at the previous moment. Meanwhile, in the process of state estimation, since the measurement data may be transmitted in an unreliable network, data packet dropout will inevitably occur, leading to a reduction in estimation accuracy. To address this undesirable situation, this paper proposes a state estimation method for nonlinear multi-sensor systems with cross-correlated noise and packet dropout compensation based on a sequential fusion framework. Firstly, a prediction compensation mechanism and a strategy based on observation noise estimation are used to update the measurement data while avoiding the noise decorrelation step. Secondly, a design step for a sequential fusion state estimation filter is derived based on an innovation analysis method. Then, a numerical implementation of the sequential fusion state estimator is given based on the third-degree spherical-radial cubature rule. Finally, the univariate nonstationary growth model (UNGM) is combined with simulation to verify the effectiveness and feasibility of the proposed algorithm.

## 1. Introduction

In recent years, the application of multi-sensor systems has gained widespread attention in the fields of signal and processing, robot localization and navigation, multi-target tracking, and navigation and guidance systems [1,2,3]. Due to the problem of network latency or limited data communication capability of sensor systems, the packet dropout and noise coupling of measurement data are inevitable [4]. This situation seriously affects the effectiveness and real-time state estimation, which leads to unreliable system networks. The noise in the system is correlated and cross-correlated, resulting from various practical scenarios. This transforms the system from an ideal, noise-independent, uncorrelated state to a complex, noise-coupled situation. The long-time noise superposition effect and dynamic influence of observed states in a common noisy environment contribute to this transformation. Therefore, the problem of state estimation with correlated noise has been a hot topic in recent years.

Kalman filtering [5] is a classical fusion algorithm for control systems, based on the assumption that process noise and observation noise are independent and uncorrelated, and it is only applicable to linear systems. In fact, common physical systems often exhibit nonlinear properties. With the development of complex control systems, a large number of state estimation methods for nonlinear systems have been proposed. First, the more common solution is based on the nonlinear function linearization of the Extended Kalman Filter (EKF) [6], and similarly the Divided Difference Filter (DDF) [7] and Central Difference Filter (CDF) [8]. Julier et al. proposed the Unscented Kalman Filter (UKF) [9] based on the traceless transformation, which obtains the desired state distribution by transforming the selected sigma points through a nonlinear transformation. Arasaratnam et al. proposed a Cubature Kalman Filter (CKF) [10] based on the third-degree spherical-radial cubature rule, which also is based on deterministic sampling to numerically approximate the integral of the nonlinear function and the Gaussian distribution in a high-dimensional scene. It has been demonstrated in the literature that the CKF has better numerical implementation stability than the UKF [11,12]. In addition, the particle filtering (PF) [13] based on the Monte Carlo sampling method, which is one of the random sampling methods, is computationally expensive and has the problem of resampling due to particle degradation, which is a tedious process.

The fusion algorithms in the field of multi-sensor information fusion [14,15] are mainly divided into three types: centralized fusion, sequential fusion, and distributed fusion [16]. The centralized fusion approach sends all observations to the data estimation center for fusion estimation at the same time step, thus requiring high computational power of the data processing center, which provides the best estimation accuracy when the sensors are in healthy working condition. However, the centralized fusion approach may produce unreliable estimations when the sensors degrade or fault. On the contrary, distributed fusion has the advantage of a parallel structure, which can detect and isolate the faulty sensors and generate local estimates based on the measurements of each sensor [17], and then send the local estimates to the fusion center to generate fusion estimates by using certain fusion rules [16]. Therefore, distributed fusion has good reliability and fault tolerance. Sequential fusion does not need to wait for all sensor data to be transmitted to the fusion center, it can process the observations in real time according to the order of their arrival at the fusion center, avoiding observation augmentation and largely reducing the computational cost.

Correlated noise is widely present in practical engineering systems, where continuous systems are discretized and synchronized with non-uniform sampling, and network systems with random delays and packet dropout can be converted to random parameterized systems with correlated noise [18]. For the study of the filtering problem of nonlinear systems with the correlated process and observation noise, the difficulty is mainly focused on how to deal with the coupling term between the random noise and the system state, which inherently causes the correlation between the process noise and the observation of the system due to the correlation of the noise space, which in turn leads to the computational complexity of the system state prediction. Yan et al. [19] focus on the noise of different sensors’ cross-correlated problems to perform optimal state estimation under linear systems. Sun et al. [20] proposed an optimal distributed fusion filter using the matrix-weighted fusion algorithm, combining the local optimal filter and cross-covariance matrix of filtering error between any two local filters. Based on this, Lin et al. [21] proposed the global optimal distributed fusion filter in the sense of linear minimum variance (LMV) based on distributed fusion as well as sequential fusion approach for linear systems. Hao et al. [22] proposed MW-CKF for nonlinear systems based on the underlying framework of cubature Kalman combined with distributed filter fusion. In order to reduce the energy loss of the system while obtaining higher estimation efficiency, Yan et al. [23] and Cheng et al. [24] proposed a sequential fusion filter based on an event-triggered mechanism. In addition, with the development of science and technology, the performance requirements of the filters are getting higher and higher in order to meet the demand for higher estimation accuracy. The presence of energy loss, data delay, and auto-correlated noise in multi-sensor network systems poses certain challenges for state estimation of complex nonlinear systems. In addition, sequential fusion can handle multi-sensor state fusion with fading measurements and noise correlation [25]. Therefore, the sequential fusion method is also applicable to the state estimation of multi-sensor nonlinear systems to solve the problem of simultaneous correlation of observation noise, where the observation noise of each sensor is correlated with the process noise of the previous moment. Therefore, in this paper, a sequential fusion approach is used for multi-sensor information fusion.

In networked systems, data packet dropout due to network congestion or sensor degradation occurs from time to time, which can easily lead to an increase in the uncertainty of system state estimation. Therefore, compensating for the lost data when the sensor measurements are lost at the current moment is also a hot issue. Currently, researchers have proposed the zero-input strategy (zero value is used for compensation), the hold-input strategy (the latest measurement received previously is used for compensation), and the measurement prediction strategy (when the measurement packet is lost at the current moment, the predicted value of the current sensor measurement is used for compensation) [26]. Zhang et al. [27] proposed a compensation strategy based on the random packet dropout problem of network control systems (NCSs) using the measurement prediction strategy. In [28], a new model with two Bernoulli random variables is used to describe the case of multiple packet dropout and one-step random delay, and the optimal linear state estimation is given in the linear least variance sense. Zhao et al. [29] use the same approach to compensate for the lost measurement data, and based on the proposed centralized and distributed fusion estimator, the optimal filter with compensating factor and the associated noise posterior covariance, new square root of error covariance factors are derived. In addition, it has been demonstrated in Ma et al. (2017) [30] that the measurement prediction strategy has higher accuracy compared to the compensation mechanisms such as the hold-input strategy.

The proposed algorithm is based on a sequential fusion framework, which ensures efficient data processing and state estimation accuracy, and is similar to the ETCKF-SF proposed by Cheng et al. [24], where the noise of each sensor is synchronously correlated [31], but the difference is that this paper adds measurement compensation and also considers the estimated amount of measurement noise to enhance the fault tolerance and robustness of the system.

Based on the above analysis, there is no relevant literature on sequential fusion filters for nonlinear multi-sensor systems with cross-correlated noise and packet dropout compensation. The main contributions of this paper are as follows: (1) an observational noise estimator based on an innovation analysis method was designed to avoid noise de-correlation; (2) based on the noise estimator, a nonlinear multi-sensor sequential fusion filter with cross-correlated noise and packet dropout compensation is designed based on the measurement prediction compensation mechanism; (3) a numerical implementation of the filter fusion algorithm proposed in this paper is carried out based on third-degree spherical-radial cubature rule deterministic sampling method.

The paper is organized as follows. In Section 2, the problem of nonlinear systems with cross-correlated noise and measurement compensation is stated. Section 3 gives the special design of the sequential fusion filter with cross-correlated noise and Section 4 gives the numerical implementation procedure of the proposed algorithm based on the third-degree spherical-radial cubature rule. Section 5 shows the simulation experiments and results analysis. Section 6 gives a summary of the paper.

Notations: $R^{n}$ represents an n-dimensional Euclidean vector space; $I_{n}$ represents an n-dimensional unit matrix; the superscript −1 and *T* represents the inverse and transpose of matrix; $\Pi_{i}\left(k+1\right)$ is a linear space spanned by $\left\{Y_{k+1}^{\left(1\right)},Y_{k+1}^{\left(2\right)},\cdots ,Y_{k+1}^{\left(i\right)}\right\}$. E[◾] denotes the mathematical expectation; (◾) is the same as the preceding neighboring term in parentheses; $\delta_{k,l}$ denotes the Kronecker Delta function; $N(\mu ,\sigma^{2})$ is the normal distribution. $\varsigma_{i}$, $\xi_{i}$, $\zeta_{i}$, $\tau_{i}$ represent the *i*th cubature point.

## 2. Problem Statement

Consider the following discrete-time nonlinear multi-sensor system with cross-correlated noise and packet dropout compensation:

(1) $$X_{k+1}=F\left(X_{k}\right)+\Gamma_{k}W_{k}$$

(2) $$Z_{k}^{\left(i\right)}=H^{\left(i\right)}\left(X_{k}\right)+V_{k}^{\left(i\right)},i=1,2,\cdots ,L$$

(3) $$Y_{k}^{\left(i\right)}=\gamma_{k}^{i}Z_{k}^{\left(i\right)}+\left(1-\gamma_{k}^{i}\right)Z_{k\left|k-1\right.}^{\left(i\right)}$$

where $X_{k}\in R^{n}$ is the state vector, $Z_{k}^{\left(i\right)}\in R^{p_{i}},i=1,2,\cdots ,L$ is the observation data of the *i*th sensor, where $p_{i}$ is the dimension of the *i*th sensor measurement data; F(◾) and H(◾) are known nonlinear function, $W_{k}\in R^{m}$ and $V_{k+1}^{\left(i\right)}\in R^{p_{i}},i=1,2,\cdots ,L$ are correlated process noise and observation noise of the *i*th sensor; $\Gamma_{k}$ is known matrix; $\gamma_{k}^{i}$ is an independent and identically distributed Bernoulli random variable, and it satisfies the probabilities $p\left(\gamma_{k}^{i}=1\right)=E\left[\gamma_{k}^{i}\right]=p_{k}^{i}$ and $p\left(\gamma_{k}^{i}=0\right)=1-E\left[\gamma_{k}^{i}\right]=1-p_{k}^{i}$, $0\leq p_{k}^{i}\leq 1$. We define Π(k+1) as the data received by the estimator up to time k+1.

(4) $$\left\{\begin{array}{c} \Pi_{i}\left(k+1\right)=\left\{Y_{k+1}^{\left(1\right)},Y_{k+1}^{\left(2\right)},\cdots ,Y_{k+1}^{\left(i\right)}\right\}\\ \Pi \left(k+1\right)=\left\{\Pi_{L}\left(0\right),\Pi_{L}\left(1\right),\cdots ,\Pi_{L}\left(k+1\right)\right\} \end{array}\right.$$

**Assumption** **A1.** *The process noise $W_{k}$ and observation noise $V_{k}^{\left(i\right)},i=1,2,\cdots ,L$ are correlated white noise satisfying the follow statistical properties: $E\left[W_{k}\right]=0$, $E\left[V_{k}^{\left(i\right)}\right]=0$, $E\left[W_{k}W_{l}^{T}\right]=Q_{k}\delta_{k,l}$, $E\left[V_{k}^{\left(i\right)}{\left(V_{l}^{\left(j\right)}\right)}^{T}\right]=R_{k}^{i,j}\delta_{k,l}$, $E\left[W_{l}{\left(V_{k}^{\left(j\right)}\right)}^{T}\right]=S_{k}^{j}\delta_{l,k-1}$. k, l denote the time step, and i, j represent the sensor serial number. Where j=1,2,⋯,L.*

**Assumption** **A2.** *The initial value $X_{0}$ is uncorrelated with $W_{k}$ and $V_{k}^{\left(i\right)},i=1,2,\cdots ,L$, and $E\left[X_{0}\right]=\mu_{0}$ and $E\left[\left(X_{0}-\mu_{0}\right){\left(X_{0}-\mu_{0}\right)}^{T}\right]=P_{0}$.*

**Assumption** **A3.** *State $X_{k}$ obeys Gaussian distribution under the condition of measurement data Π(k−1)*

(5) $$p\left(X_{k}|\Pi \left(k-1\right)\right)=N\left(X_{k};{\hat{X}}_{k|k-1}^{0},P_{k|k-1}^{X^{0}}\right),k\geq 1$$

In sequential fusion, the global state estimate at the current moment becomes the initial state at the next moment after iteration so that the state expectation under the condition of measure Π(k−1) obeys a Gaussian distribution of $N(X_{k};{\hat{X}}_{k|k-1}^{0},P_{k|k-1}^{X^{0}})$. The superscript 0 indicates the initial or a priori state.

**Assumption** **A4.** *When l≥i, state $X_{k+1}^{l}$ obeys Gaussian distribution under the condition of measurement data $\left\{\Pi \left(k\right),\Pi_{i}\left(k+1\right)\right\}$*

(6) $$p\left(X_{k+1}^{l}|\Pi \left(k\right),\Pi_{i}\left(k+1\right)\right)=N\left(X_{k+1};{\hat{X}}_{k+1}^{l|i},P_{k+1|k+1}^{l|i}\right)=N\left(X_{k+1};{\hat{X}}_{k+1}^{i},P_{k+1|k+1}^{X^{i}}\right),k\geq 0$$

## 3. Design of Sequential Filter for Nonlinear Multi-Sensor with Correlated Noise and Dropout Packet Compensation

Since the dropout of measurement packets and correlated noise problems in multi-sensor systems are considered, we incorporate measurement compensation to ensure the completeness of communication data information and use a sequential fusion framework, combined with noise synchronization correlation, to estimate the state of the multi-sensor system.

Let ${\hat{X}}_{k+1}^{i}=E\left[X_{k+1}\left|\Pi \left(k\right),\Pi_{i}\left(k+1\right)\right.\right]$, ${\hat{X}}_{k+1}^{-}=E\left[X_{k+1}\left|\Pi (k)\right.\right]$, $V_{k+1\left|k+1\right.}^{\left(i\left|0\right.\right)}=E\left[V_{k+1}^{\left(i\right)}\left|\Pi (k)\right.\right]$=0 and $V_{k+1\left|k+1\right.}^{\left(i\left|i-1\right.\right)}=E\left[V_{k+1}^{\left(i\right)}\left|\Pi \left(k\right),\Pi_{i-1}\left(k+1\right)\right.\right]$. Based on measurements Π(k+1), we derive the sequential fusion filter ${\hat{X}}_{k+1}^{s}$ and its covariance matrix $P_{k+1\left|k+1\right.}^{X^{s}}$.

**Theorem** **1.** *For systems* (1)–(3), *under the conditions of Assumptions 1–4, we have the sequential fusion state filter with mean ${\hat{X}}_{k+1}^{i}$ and covariance $P_{k+1\left|k+1\right.}^{X^{i}}$ and its initial values $\mu_{0}={\hat{X}}_{0|-1}$ and $P_{0}=P_{0|-1}$.*

The sequential filter ${\hat{X}}_{k+1}^{i}$ and its filtering error covariance matrix $P_{k+1\left|k+1\right.}^{X^{i}}$ for the state are calculated as follows:

(7) $${\hat{X}}_{k+1}^{i}={\hat{X}}_{k+1}^{i-1}+M_{k+1}^{X^{i}}\varepsilon_{i}\left(k+1\right)$$

(8) $$P_{k+1\left|k+1\right.}^{X^{i}}=P_{k+1\left|k+1\right.}^{X^{i-1}}-M_{k+1}^{X^{i}}Q_{\varepsilon }^{i}\left(k+1\right){\left(M_{k+1}^{X^{i}}\right)}^{T}$$

where the expressions of innovation $\varepsilon_{i}\left(k+1\right)$, innovation covariance matrix $Q_{\varepsilon }^{i}\left(k+1\right)$, and filter gain matrix $M_{k+1}^{X^{i}}$ of the *i*th sensor at time k+1 are

(9) $$\varepsilon_{i}\left(k+1\right)=\gamma_{k+1}^{i}Z_{k+1}^{\left(i\right)}+\left(p_{k+1}^{i}-\gamma_{k+1}^{i}\right)Z_{k+1\left|k\right.}^{\left(i\right)}-p_{k+1}^{i}Z_{k+1\left|k+1\right.}^{\left(i\left|i-1\right.\right)}$$

(10) $$\begin{array}{cc} \; & Q_{\varepsilon }^{i}\left(k+1\right)=p_{k+1}^{i}E\left[\left(Z_{k+1}^{\left(i\right)}\right){\left(◾\right)}^{T}\left|\Pi \left(k\right),\Pi_{i-1}\left(k+1\right)\right.\right]\\ \; & +p_{k+1}^{i}\left(1-p_{k+1}^{i}\right)Z_{k+1\left|k\right.}^{\left(i\right)}{\left(Z_{k+1\left|k\right.}^{\left(i\right)}\right)}^{T}+p_{k+1}^{i}\left(p_{k+1}^{i}-1\right)Z_{k+1\left|k+1\right.}^{\left(i\left|i-1\right.\right)}{\left(Z_{k+1\left|k\right.}^{\left(i\right)}\right)}^{T}\\ \; & +{\left(p_{k+1}^{i}\left(p_{k+1}^{i}-1\right)Z_{k+1\left|k+1\right.}^{\left(i\left|i-1\right.\right)}{\left(Z_{k+1\left|k\right.}^{\left(i\right)}\right)}^{T}-{\left(p_{k+1}^{i}\right)}^{2}Z_{k+1\left|k+1\right.}^{\left(i\left|i-1\right.\right)}{\left(Z_{k+1\left|k+1\right.}^{\left(i\left|i-1\right.\right)}\right)}^{T}\right)}^{T} \end{array}$$

(11) $$M_{k+1}^{X^{i}}=P_{k+1\left|k+1\right.}^{X^{i-1}Y^{i\left|i-1\right.}}{\left(Q_{\varepsilon }^{i}\left(k+1\right)\right)}^{-1}$$

where the covariance matrix between state estimation error and innovation is:

(12) $$\begin{array}{cc} \; & P_{k+1|k+1}^{X^{i-1}Y^{i|i-1}}=p_{k+1}^{i}{\int X_{k+1}\left(H^{\left(i\right)}\left(X_{k+1}\right)\right)}^{T}N\left(X_{k+1};{\hat{X}}_{k+1}^{i-1},P_{k+1|k+1}^{X^{i-1}}\right)dX_{k+1}\\ \; & +p_{k+1}^{i}{\int X_{k+1}\left(V_{k+1}^{\left(i\right)}\right)}^{T}N\left(\left[\begin{array}{c} X_{k+1}\\ V_{k+1}^{\left(i\right)} \end{array}\right];\left[\begin{array}{c} {\hat{X}}_{k+1}^{i-1}\\ V_{k+1}^{\left(i|i-1\right)} \end{array}\right],\Psi_{1}\right)d\left[\begin{array}{c} X_{k+1}\\ V_{k+1}^{\left(i\right)} \end{array}\right]-p_{k+1}^{i}{\hat{X}}_{k+1}^{i-1}{\left(Z_{k+1\left|k+1\right.}^{\left(i|i-1\right)}\right)}^{T} \end{array}$$

(13) $$\Psi_{1}=\left[\begin{array}{cc} P_{k+1|k+1}^{X^{i-1}} & P_{k+1|k+1}^{X^{i-1}V^{i|i-1}}\\ {\left(P_{k+1|k+1}^{X^{i-1}V^{i|i-1}}\right)}^{T} & P_{k+1|k+1}^{V^{i|i-1}} \end{array}\right]$$

(14) $$\begin{array}{cc} \; & E\left[\left(Z_{k+1}^{\left(i\right)}\right){\left(\cdot \right)}^{T}|\Pi \left(k\right),\Pi_{i-1}\left(k+1\right)\right]\\ = & \int H^{\left(i\right)}\left(X_{k+1}\right){\left(H^{\left(i\right)}\left(X_{k+1}\right)\right)}^{T}N\left(X_{k+1};{\hat{X}}_{k+1}^{i-1},P_{k+1|k+1}^{X^{i-1}}\right)dX_{k+1}\\ \; & +\left(\int H^{\left(i\right)}\left(X_{k+1}{\left(V_{k+1}^{\left(i\right)}\right)}^{T}N\left(\left[\begin{array}{c} X_{k+1}\\ V_{k+1}^{\left(i\right)} \end{array}\right];\left[\begin{array}{c} {\hat{X}}_{k+1}^{i-1}\\ {\hat{V}}_{k+1}^{\left(i)i-1\right)} \end{array}\right],\Psi_{1}\right)d\left[\begin{array}{c} X_{k+1}\\ V_{k+1}^{\left(i\right)} \end{array}\right]\right)+{\left(\cdot \right)}^{T}+R_{k+1}^{i}\right. \end{array}$$

(15) $$Z_{k+1\left|k\right.}^{\left(i\right)}=E\left[Z_{k+1}^{\left(i\right)}\left|\Pi (k)\right.\right]=\int H^{\left(i\right)}\left(X_{k+1}\right)N\left(X_{k+1};{\hat{X}}_{k+1\left|k\right.}^{0},P_{k+1\left|k\right.}^{X^{0}}\right)dX_{k+1}$$

The predicted estimate of the sensor observation is:

(16) $$Z_{k+1\left|k+1\right.}^{\left(i\left|i-1\right.\right)}=\int H^{\left(i\right)}\left(X_{k+1}\right)N\left(X_{k+1};{\hat{X}}_{k+1}^{i-1},P_{k+1\left|k+1\right.}^{X^{i-1}}\right)dX_{k+1}+V_{k+1\left|k+1\right.}^{\left(i\left|i-1\right.\right)}$$

Substituting the above Equations (12)–(16) into Equations (10) and (11) gives $Q_{\varepsilon }^{i}\left(k+1\right)$ and $M_{k+1}^{X^{i}}$.

The estimated value of observation noise is:

(17) $$V_{k+1\left|k+1\right.}^{\left(i\left|i-1\right.\right)}=\sum_{l=1}^{i-1}M_{k+1}^{V^{i\left|l\right.}}\varepsilon_{l}\left(k+1\right)$$

The calculation of gain matrix $M_{k+1}^{V^{i\left|l\right.}}\;l=1,2,\cdots ,i-1$, cross covariance matrix $P_{k+1\left|k+1\right.}^{X^{i-1}V^{i\left|i-1\right.}}$ between state estimation error and observation noise is as follows:

(18) $$M_{k+1}^{V^{i\left|l\right.}}=p_{k+1}^{l}\left(E\left[V_{k+1}^{\left(i\right)}{\left(Z_{k+1}^{\left(l\right)}\right)}^{T}\right]-E\left[V_{k+1}^{\left(i\right)}{\left(Z_{k+1\left|k+1\right.}^{\left(l\left|l-1\right.\right)}\right)}^{T}\right]\right){\left(Q_{\varepsilon }^{l}\left(k+1\right)\right)}^{-1}$$

(19) $$P_{k+1\left|k+1\right.}^{X^{l-1}V^{i\left|l-1\right.}}=P_{k+1\left|k+1\right.}^{X^{l-2}V^{i\left|l-2\right.}}-M_{k+1}^{X^{l-1}}Q_{\varepsilon }^{l-1}\left(k+1\right){\left(M_{k+1}^{V^{i\left|l-1\right.}}\right)}^{T}$$

where

(20) $$\begin{array}{cc} \; & E\left[V_{k+1}^{\left(i\right)}{\left(Z_{k+1}^{\left(i-1\right)}\right)}^{T}|\Pi \left(k\right),\Pi_{i-2}\left(k+1\right)\right]\\ \; & =\int V_{k+1}^{\left(i\right)}{\left(H^{\left(i-1\right)}\left(X_{k+1}\right)\right)}^{T}N\left(\left[\begin{array}{c} X_{k+1}\\ V_{k+1}^{\left(i\right)} \end{array}\right];\left[\begin{array}{c} {\hat{X}}_{k+1|k+1}^{\left(i-2\right)}\\ V_{k+1|k+1}^{\left(i|-2\right)} \end{array}\right],\Psi_{2}\right)d\left[\begin{array}{c} X_{k+1}\\ V_{k+1}^{\left(i\right)} \end{array}\right]+R_{k+1}^{i,i-1} \end{array}$$

(21) $$\Psi_{2}=\left[\begin{array}{cc} P_{k+1\left|k+1\right.}^{X^{i-2}} & P_{k+1\left|k+1\right.}^{X^{i-2}V^{i\left|i-2\right.}}\\ {\left(P_{k+1\left|k+1\right.}^{X^{i-2}V^{i\left|i-2\right.}}\right)}^{T} & P_{k+1\left|k+1\right.}^{V^{i\left|i-2\right.}} \end{array}\right]$$

(22) $$E\left[V_{k+1}^{\left(i\right)}{\left(Z_{k+1\left|k+1\right.}^{\left(i-1\left|i-2\right.\right)}\right)}^{T}\left|\Pi \left(k\right),\Pi_{i-2}\left(k+1\right)\right.\right]=V_{k+1}^{\left(i\left|i-2\right.\right)}{\left(Z_{k+1\left|k+1\right.}^{\left(i-1\left|i-2\right.\right)}\right)}^{T}$$

where the covariance matrix between the state estimation error and the observation noise is:

(23) $$P_{k+1\left|k+1\right.}^{X^{l-1}V^{i\left|l-1\right.}}=S_{i}\left(k\right)-\sum_{p=1}^{l-1}M_{k+1}^{X^{p}}Q_{\varepsilon }^{p}\left(k+1\right){\left(M_{k+1}^{V^{i\left|p\right.}}\right)}^{T}$$

The cross-covariance matrix between the observation noise and its estimation error is

(24) $$P_{k+1\left|k+1\right.}^{V^{i\left|l-1\right.}}=R_{i}\left(k+1\right)-\sum_{p=1}^{l-1}M_{k+1}^{V^{i\left|p\right.}}Q_{\varepsilon }^{p}\left(k+1\right){\left(M_{k+1}^{V^{i\left|p\right.}}\right)}^{T}$$

Then, considering the recursive framework of the sequential fusion filter, the state estimation and estimation error covariance matrix at instant k+1 are

(25) $$\left\{\begin{array}{c} {\hat{X}}_{k+1}^{s}={\hat{X}}_{k+1}^{L}\\ P_{k+1|k+1}^{X^{s}}=P_{k+1|k+1}^{X^{L}} \end{array}\right.$$

Then, the predictor of sequential fusion is

(26) $$\begin{array}{cc} \; & {\hat{X}}_{k+1}^{-}=E\left[X_{k+1}\left|\Pi (k)\right.\right]\\ \; & =\int F\left(X_{k}\right)N\left(X_{k};{\hat{X}}_{k}^{L},P_{k\left|k\right.}^{X^{L}}\right)dX_{k} \end{array}$$

(27) $$P_{k+1|k+1}^{X^{-}}=\int F\left(X_{k}\right)F^{T}\left(X_{k}\right)N\left(X_{k};{\hat{X}}_{k}^{L},P_{k|k}^{X^{L}}\right)dX_{k}-{\hat{X}}_{k+1}^{-}{\left({\hat{X}}_{k+1}^{-}\right)}^{T}+Q_{k}$$

Then, ${\hat{X}}_{k+1}^{0}$ and $P_{k+1\left|k+1\right.}^{X^{0}}$ are calculated as follows

(28) $$\left\{\begin{array}{c} {\hat{X}}_{k+1}^{0}={\hat{X}}_{k+1}^{-}\\ P_{k+1|k+1}^{X^{0}}=P_{k+1|k+1}^{X^{-}} \end{array}\right.$$

The detailed derivation process of the above formula can be found in Appendix A.

In the table below, the implementation steps of Algorithm 1 are summarized.
**Algorithm 1:** Iterative steps of the sequential fusion algorithmInput: Sensor measurement data $Z_{k}^{\left(i\right)}$ for the *k*th time and sensor packet dropout rate $p_{k}^{i}$.Output: The posterior estimate ${\hat{X}}_{k|k}$ of the state and the variance $P_{k|k}^{X}$ of the state error.Initialization: Setting the initial state ${\hat{X}}_{0}^{-}=\mu_{0}$, the variance $P_{0}^{-}=P_{0}$, the process noise variance $Q_{k}=\sigma_{W}^{2}$, the observation noise variance $R_{i}\left(k\right)$ and the covariance $S_{i,j}\left(k\right)$ between them, and the sensor packet dropout rate $E\left[\gamma_{k}^{i}=1\right]=p_{k}^{i}$.Stateone−stepprediction: Define $V_{k}^{\left(1|0\right)}=0$ and calculate ${\hat{X}}_{k+1}^{0}$, $P_{k+1\left|k+1\right.}^{X^{0}}$ according to Equations (26) and (27).Measurementupdate: Obtain the measurement information $Z_{k+1}^{\left(i\right)}$ for each sensor and calculate the innovation $\varepsilon_{i}\left(k+1\right)$ according to Equation (9), which is then calculated by the following process:     For i:=1 to *L* do        Set $P_{k+1|k+1}^{X^{0}V^{i|0}}=S_{i}\left(k+1\right)$, $P_{k+1|k+1}^{V^{i|0}V^{1|0}}=R_{i,1}\left(k+1\right)$.        If i>1            For l:=1 to i−1 do               If l>1               Calculate $P_{k+1\left|k+1\right.}^{X^{l-1}V^{i\left|l-1\right.}}$ and $P_{k+1\left|k+1\right.}^{V^{i\left|l-1\right.}}$ according to (23) and (24), respectively.               End               Calculate $M_{k+1}^{V^{i\left|l\right.}}$ according to (18).            End            Calculate $V_{k+1\left|k+1\right.}^{\left(i\left|i-1\right.\right)}$ according to (17).        End        Calculate $Q_{\varepsilon }^{i}\left(k+1\right)$ according to (10).        Calculate $P_{k+1|k+1}^{X^{i-1}Y^{i|i-1}}$ according to (12).        Calculate ${\hat{X}}_{k+1}^{i}$, $P_{k+1\left|k+1\right.}^{X^{i}}$ according to (7) and (8).     EndStateiteration: Calculate ${\hat{X}}_{k+1}^{s}$ and $P_{k+1\left|k+1\right.}^{X^{s}}$ by (25); Calculate ${\hat{X}}_{k+1}^{-}$ and $P_{k+1\left|k+1\right.}^{X^{-}}$ according to (26) and (27).Finally, let k=k+1. Return to Stateone−stepprediction.

## 4. Numerical Implementation Based on the Third-Degree Spherical-Radial Cubature Rule

Based on the third-degree spherical-radial cubature rule, the sequential fusion algorithm for nonlinear filtering with cross-correlated noise and measurement compensation in this section can be implemented recursively by the following numerical calculation. Assuming all the quantities before the k+1 time are known and the states of the previous local filters, i.e., ${\hat{X}}_{k+1}^{l-1}$, $P_{k+1|k+1}^{X^{l-1}}$, and $Q_{\varepsilon }^{l-1}\left(k+1\right)$, are known, the corresponding numerical implementation is detailed in the following flow.

### 4.1. Estimation Calculation of Observation Noise

We define α as follows:

(29) $$\alpha =\left[\begin{array}{c} {\hat{X}}_{k+1}^{l-1}\\ V_{k+1}^{i|l-1} \end{array}\right]$$

Cholesky factorization:

(30) $$\Psi_{1}=\Phi_{1}{\left(\Phi_{1}\right)}^{T}$$

Calculation of cubature points:

(31) $$\Theta_{t,k+1|k+1}^{\alpha }=\left[\begin{array}{c} \Theta_{t,k+1|k+1}^{x}\\ \Theta_{t,k+1|k+1}^{V} \end{array}\right]=\Phi_{1}\xi_{t}+\alpha ,t=1,2,\cdots ,2\left(n+p_{i}\right)$$

Propagate the cubature points:

(32) $${\overset{⌣}{\lambda }}_{t,k+1|k+1}^{X}=H\left(\Theta_{t,k+1|k+1}^{X}\right),t=1,2,\cdots ,2\left(n+p_{i}\right)$$

Then, the noise gain has the following result:

(33) $$\delta 1=E\left[V_{k+1}^{\left(i\right)}{\left(Z_{k+1}^{\left(l\right)}\right)}^{T}\right]=\frac{1}{2(n+p_{i})}\sum_{t=1}^{2(n+p_{i})}\Theta_{t,k+1|k+1}^{V}{\left({\overset{⌣}{\lambda }}_{t,k+1|k+1}^{X}\right)}^{T}+R_{k+1}^{i,i-1}$$

(34) $$M_{k+1}^{V^{i\left|l\right.}}=p_{k+1}^{l}\left(\delta 1-V_{k+1}^{\left(i|l-1\right)}{\left(Z_{k+1\left|k+1\right.}^{\left(l\left|l-1\right.\right)}\right)}^{T}\right){\left(Q_{\varepsilon }^{l}\left(k+1\right)\right)}^{-1}$$

$Q_{\varepsilon }^{i-1}\left(k+1\right)$ is a known volume, the cross-covariance $P_{k+1\left|k+1\right.}^{X^{l-1}V^{i\left|l-1\right.}}$ can be derived from the (33) and (34). In the recursive process, the cross-covariance $P_{k+1\left|k+1\right.}^{V^{i\left|i-1\right.}}$ between the observation noise and its estimation error can also be calculated by using the above results to obtain the Equation (24).

### 4.2. Estimation Calculation of System State

Cholesky factorization:

(35) $$\Psi_{2}=\Phi_{2}{\left(\Phi_{2}\right)}^{T}$$

(36) $$P_{k+1|k+1}^{X^{i-1}}=\eta_{k+1|k+1}{\left(\eta_{k+1|k+1}\right)}^{T}$$

(37) $$P_{k+1|k}^{X^{0}}=\Xi_{k+1|k}{\left(\Xi_{k+1|k}\right)}^{T}$$

Calculation of cubature points:

(38) $$\Theta_{t,k+1|k+1}^{\alpha }=\left[\begin{array}{c} \Theta_{t,k+1|k+1}^{X}\\ \Theta_{t,k+1|k+1}^{V} \end{array}\right]=\Phi_{2}\xi_{t}+\left[\begin{array}{c} {\hat{X}}_{k+1}^{i-1}\\ V_{k+1}^{i|i-1} \end{array}\right],t=1,2,\cdots ,2\left(n+p_{i}\right)$$

(39) $$\Upsilon_{\bar{t},k+1|k+1}^{X}=\eta_{k+1|k+1}\zeta_{\bar{t}}+{\hat{X}}_{k+1}^{i-1},\bar{t}=1,2,\cdots ,2n$$

(40) $$\Delta_{\bar{t},k+1|k}^{X^{0}}=\Xi_{k+1|k}\varsigma_{\bar{t}}+{\hat{X}}_{k+1|k}^{0},\bar{t}=1,2,\cdots ,2n$$

Propagate the cubature points:

(41) $${\overset{⌣}{\lambda }}_{t,k+1|k+1}^{X}=H\left(\Theta_{t,k+1|k+1}^{X}\right),t=1,2,\cdots ,2\left(n+p_{i}\right)$$

(42) $$\lambda_{\bar{t},k+1|k+1}^{X}=H\left(\Upsilon_{\bar{t},k+1|k+1}^{X}\right)\bar{t}=1,2,\cdots ,2n$$

(43) $${\tilde{\lambda }}_{\bar{t},k+1|k}^{X^{0}}=H\left(\Delta_{\bar{t},k+1|k}^{X^{0}}\right),\bar{t}=1,2,\cdots ,2n$$

Therefore, the innovation and the cross-covariance matrix of the state error and the measurement error are:

(44) $$\varepsilon_{i}\left(k+1\right)=\gamma_{k+1}^{i}Z_{k+1}^{\left(i\right)}+\left(p_{k+1}^{i}-\gamma_{k+1}^{i}\right)\delta 4-p_{k+1}^{i}\delta 3$$

(45) $$\begin{array}{cc} P_{k+1|k+1}^{X^{i-1}Y_{i-1}^{i-1}} & =p_{k+1}^{i}\frac{1}{2n}\sum_{\bar{t}=1}^{2n}\Upsilon_{\bar{t},k+1|k+1}^{X}{\left(\lambda_{\bar{t},k+1|k+1}^{X}\right)}^{T}\\ \; & +p_{k+1}^{i}\frac{1}{2\left(n+p_{i}\right)}\sum_{t=1}^{2\left(n+p_{i}\right)}\Theta_{t,k+1|k+1}^{X}{\left(\Theta_{t,k+1|k+1}^{V}\right)}^{T}-p_{k+1}^{i}{\hat{X}}_{k+1}^{i-1}{\left(\delta 4\right)}^{T} \end{array}$$

(46) $$\begin{array}{cc} \delta 2 & =E\left[\left(Z_{k+1}^{\left(i\right)}\right){\left(\cdot \right)}^{T}|\Pi \left(k\right),\Pi_{i-1}\left(k+1\right)\right]\\ \; & =\frac{1}{2n}\sum_{\bar{t}=1}^{2n}\lambda_{\bar{t},k+1|k+1}^{X}{\left(\lambda_{\bar{t},k+1|k+1}^{X}\right)}^{T}+\frac{1}{2\left(n+p_{i}\right)}\sum_{t=1}^{2\left(n+p_{i}\right)}\lambda_{t,k+1|k+1}^{X}{\left(\Theta_{t,k+1|k+1}^{V}\right)}^{T}\\ \; & +{\left(\frac{1}{2\left(n+p_{i}\right)}\sum_{t=1}^{2\left(n+p_{i}\right)}\lambda_{t,k+1|k+1}^{X}{\left(\Theta_{t,k+1|k+1}^{V}\right)}^{T}\right)}^{T}+R_{k+1}^{i} \end{array}$$

(47) $$\delta 3=Z_{k+1\left|k+1\right.}^{\left(i\left|i-1\right.\right)}=\frac{1}{2n}\sum_{\bar{t}=1}^{2n}\lambda_{\bar{t},k+1|k+1}^{X}+V_{k+1\left|k+1\right.}^{\left(i\left|i-1\right.\right)}$$

(48) $$\delta 4=Z_{k+1\left|k\right.}^{\left(i\right)}=\frac{1}{2n}\sum_{\bar{t}=1}^{2n}{\tilde{\lambda }}_{\bar{t},k+1|k}^{X^{0}}$$

The measurement error covariance is:

(49) $$\begin{array}{cc} Q_{\varepsilon }^{i}\left(k+1\right) & =p_{k+1}^{i}\delta 2+p_{k+1}^{i}\left(1-p_{k+1}^{i}\right)\delta 4{\left(\delta 4\right)}^{T}+p_{k+1}^{i}\left(p_{k+1}^{i}-1\right)\delta 3{\left(\delta 4\right)}^{T}\\ \; & +{\left(p_{k+1}^{i}\left(p_{k+1}^{i}-1\right)\delta 3{\left(\delta 4\right)}^{T}-{\left(p_{k+1}^{i}\right)}^{2}\delta 3{\left(\delta 3\right)}^{T}\right)}^{T} \end{array}$$

as a result, $M_{k+1}^{X^{i}}$ can be obtained using the above calculation, while Equations (7) and (8) can be calculated from Equations (44), (45), and (49) as the final result, where $p_{k+1}^{i}$ is the probability of dropout of measurement data.

The above steps are repeated until all sensor data are calculated at time k+1. The state data obtained from the fusion center is used for one-step state prediction.

### 4.3. One-Step Prediction Estimate and One-Step Prediction Covariance Matrix of State

Cholesky factorization:

(50) $$P_{k+1\left|k+1\right.}^{X^{L}}=S_{k+1\left|k+1\right.}^{X}{\left(S_{k+1\left|k+1\right.}^{X}\right)}^{T}$$

Calculation of cubature points:

(51) $${\overset{⌢}{\Upsilon }}_{\bar{t},k+1|k+1}^{X}=S_{k+1\left|k+1\right.}^{X}\tau_{\bar{t},k+1|k+1}+{\hat{X}}_{k+1}^{L}\bar{t}=1,2,\cdots ,2n$$

Propagate the cubature points:

(52) $${\overset{⌢}{\lambda }}_{\bar{t},k+1|k+1}^{X}=F\left({\overset{⌢}{\Upsilon }}_{\bar{t},k+1|k+1}^{X}\right)\bar{t}=1,2,\cdots ,2n$$

Then, the value of the one-step state prediction is:

(53) $${\hat{X}}_{k+1}^{0}={\hat{X}}_{k+1}^{-}=\frac{1}{2n}\sum_{\bar{t}=1}^{2n}{\overset{⌢}{\lambda }}_{\bar{t},k+1|k+1}^{\hat{X}}$$

(54) $$P_{k+1\left|k+1\right.}^{X^{0}}=P_{k+1\left|k+1\right.}^{X^{-}}=\frac{1}{2n}\sum_{\bar{t}=1}^{2n}{\overset{⌢}{\lambda }}_{\bar{t},k+1|k+1}^{X}{\left({\overset{⌢}{\lambda }}_{\bar{t},k+1|k+1}^{X}\right)}^{T}-{\hat{X}}_{k+1}^{-}{\left({\hat{X}}_{k+1}^{-}\right)}^{T}+Q_{k}$$

The above is performed after all sensor data processing is completed, and the posterior estimate of the current moment is used as the a priori state data for the next moment.

## 5. Simulation

This paper conducts CKF numerical simulation experiments based on the filter algorithm proposed in Section 3 and uses the univariate nonstationary growth model (UNGM) to test the effectiveness of the proposed algorithm.We assume that the entire system includes two sensors and the state and measurement equations of the system can be described as follows:

(55) $$X_{k}=0.5X_{k-1}+25\frac{X_{k-1}}{1+X_{k-1}^{2}}+8\cos\left(1.2\left(k-1\right)\right)+W_{k-1}$$

(56) $$Z_{k}^{\left(1\right)}=\frac{X_{k}^{2}}{20}+V_{1}\left(k\right)$$

(57) $$Z_{k}^{\left(2\right)}=\frac{X_{k}^{2}}{20}+V_{2}\left(k\right)$$

In the above equation, (56) and (57) are the measurement models for each of the two sensors, and the superscripts 1,2 are the *i*th sensor. $W_{k}$ is a Gaussian white noise obeying a mean of zero and a variance of $Q_{k}=\sigma_{W}^{2}$. In addition, the process noise is related to the measurement noise of each sensor, satisfying the following relationship:

(58) $$V_{1}\left(k\right)=\eta_{1}\left(k\right)+\beta_{1}W\left(k-1\right)$$

(59) $$V_{2}\left(k\right)=\eta_{2}\left(k\right)+\beta_{2}W\left(k-1\right)$$

The $W_{k}$ and Gaussian white noise $\eta_{i}\left(k\right)∼N\left(0,\sigma_{\eta_{i}}^{2}\right)$ are independent of each other. The variance and covariance of the observation noise for different sensors are $R_{i}\left(k\right)=\sigma_{\eta_{i}}^{2}+\beta_{i}\sigma_{W}^{2}$, $R_{i,j}\left(k\right)=\beta_{i}\beta_{j}\sigma_{W}^{2},i\neq j$ and $S_{i}\left(k\right)=\beta_{i}\sigma_{W}^{2}$, respectively. In the experiments, the role of the measurement prediction compensation strategy needs to be taken into account, and the measurement prediction compensation model is defined as follows:

(60) $$Y_{k}^{\left(1\right)}=\gamma_{k}^{1}Z_{k}^{\left(1\right)}+\left(1-\gamma_{k}^{1}\right)Z_{k|k-1}^{\left(1\right)}$$

(61) $$Y_{k}^{\left(2\right)}=\gamma_{k}^{2}Z_{k}^{\left(2\right)}+\left(1-\gamma_{k}^{2}\right)Z_{k|k-1}^{\left(2\right)}$$

where $\gamma_{k}^{1}$ and $\gamma_{k}^{2}$ are the independent and identically distributed Bernoulli random variable for the two sensors at the *k*th time step. We define the 70 as the estimated length of state X and a single time sampling step T=1. The error of 100 Monte Carlo runs and the root mean square error (RMSE) can be defined as follows:

(62) $$Error=\frac{1}{N}\sum_{n=1}^{N}\left(X_{k}^{\left(n\right)}-{\hat{X}}_{k|k}^{\left(n\right)}\right),1\leq k\leq 70$$

(63) $$RMSE=\sqrt{\frac{1}{N}\sum_{n=1}^{N}{\left(X_{k}^{\left(n\right)}-{\hat{X}}_{k|k}^{\left(n\right)}\right)}^{2}},1\leq k\leq 70$$

where $X_{k}^{\left(n\right)}$ and ${\hat{X}}_{k|k}^{\left(n\right)}$ are the ground truth of the state and the estimated state of the *k*th epoch at the *n*th Monte Carlo run, respectively. N=100 is the number of Monte Carlo runs.

Set $\sigma_{W}^{2}=5$, $\sigma_{\eta_{1}}^{2}=2.46$, $\sigma_{\eta_{2}}^{2}=8.75$, $\beta_{1}=0.8$, $\beta_{2}=0.5$ and covariance $R_{1,2}=0.19$. The initial value of the state, as well as the initial variance, are $\mu_{0}=0.3$, $P_{0}=5$. In order to investigate the effect of state estimation under packet dropout compensation, we set up multiple comparison experiments under different communication rates based on different communication rates, and also compare the local estimation results with the sequential estimation results.

The local estimation is the fusion estimate of half of the total number of sensors, and sequential estimation is the fusion estimate of all sensors.

**Figure 1:**
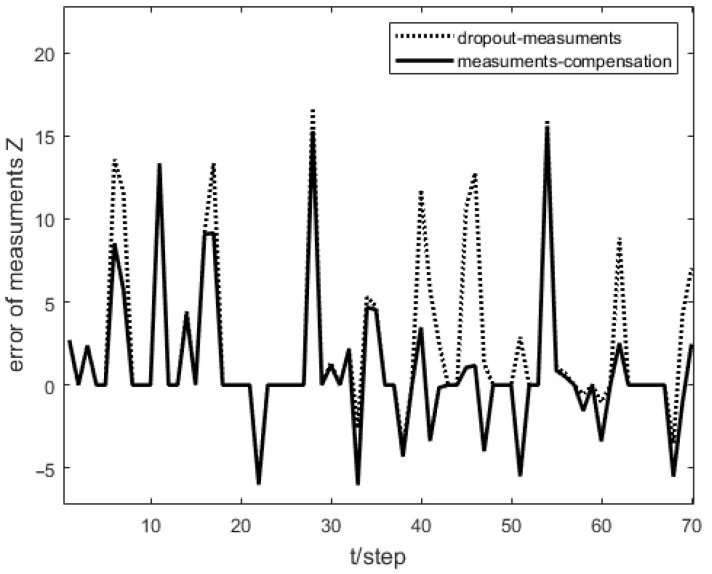
Measurement error of sensor 1 in the case of packet dropout and compensation mechanism with $p_{k}^{1}=0.4$.

**Figure 2:**
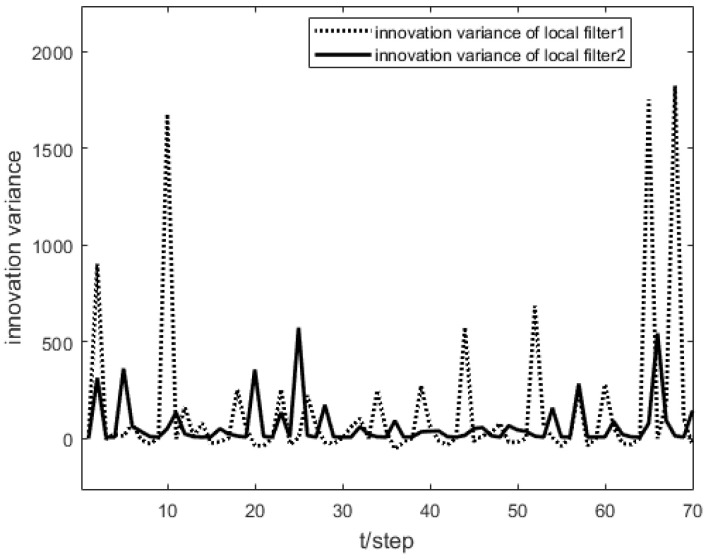
Innovation variance of two local filters with $p_{k}^{1}=0.4,p_{k}^{2}=0.7$.

**Figure 3:**
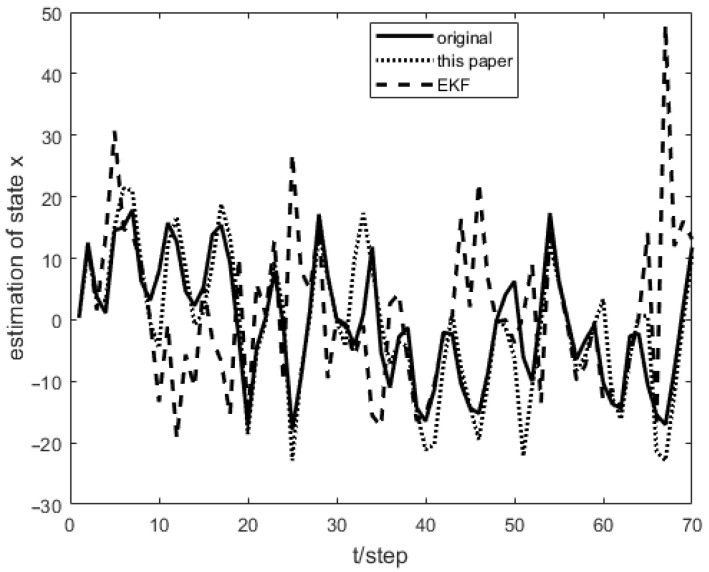
Comparison of the algorithm of this paper and EKF with $p_{k}^{1}=0.4,p_{k}^{2}=0.7$.

**Figure 4:**
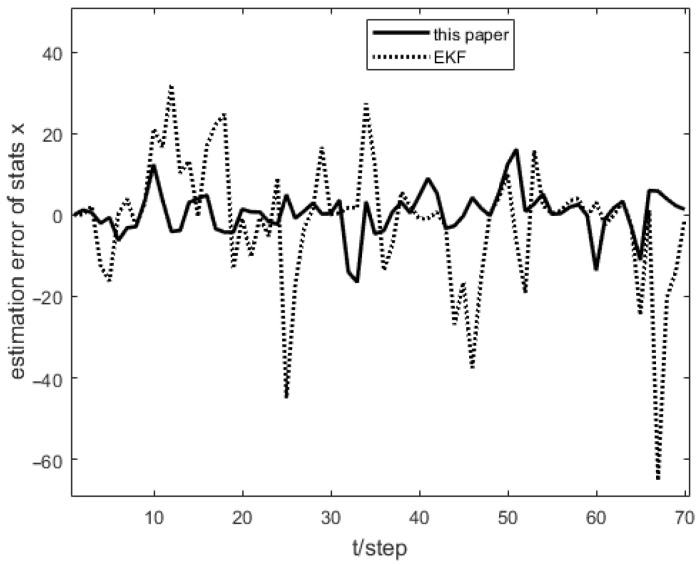
Error of proposed algorithm and EKF with $p_{k}^{1}=0.4,p_{k}^{2}=0.7$.

**Figure 5:**
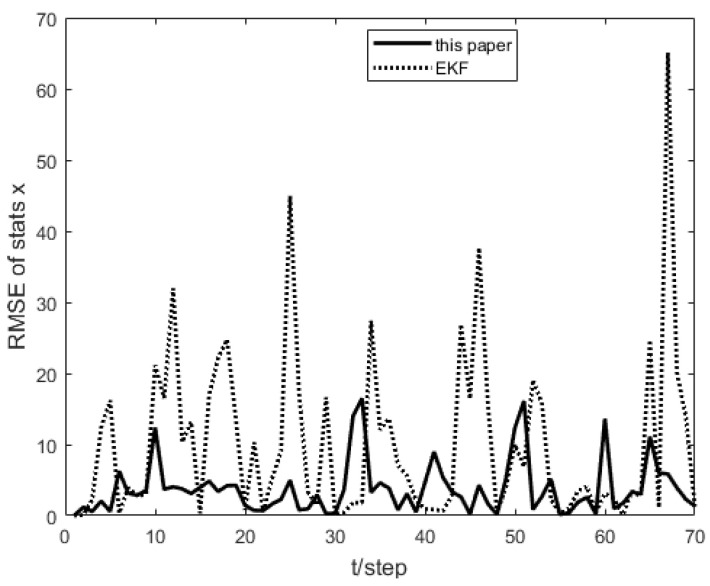
RMSE of proposed algorithm and EKF with $p_{k}^{1}=0.4,p_{k}^{2}=0.7$.

**Figure 6:**
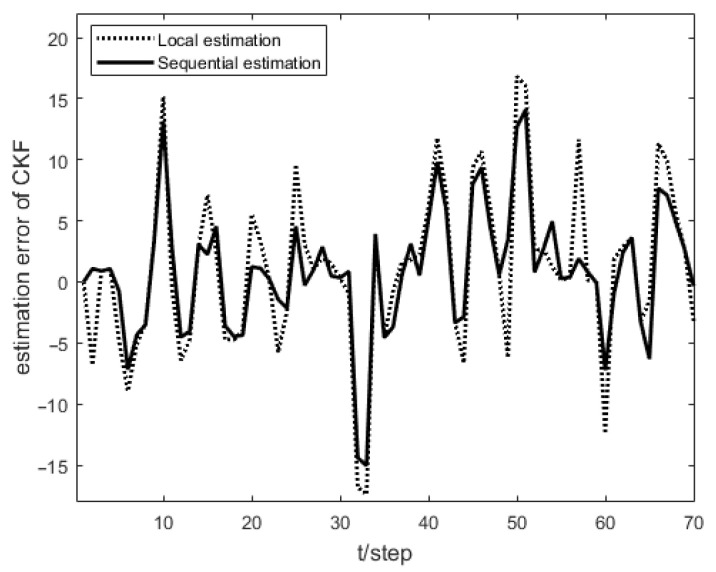
Error of partial sensors fusion and all sensors fusion with $p_{k}^{1}=0.4,p_{k}^{2}=0.7$.

**Figure 7:**
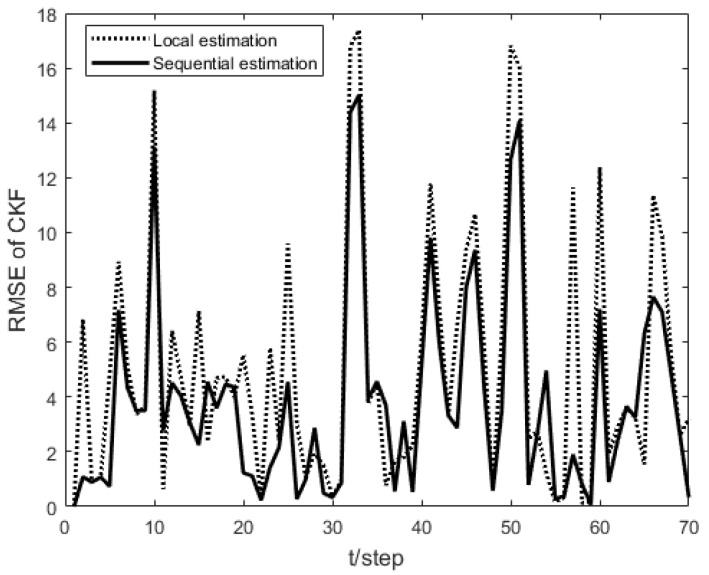
RMSE of partial sensors fusion and all sensors fusion with $p_{k}^{1}=0.4,p_{k}^{2}=0.7$.

As shown in Figure 1, the measurement prediction compensation mechanism can reduce measurement errors to a certain level and prevent observation scattering due to packet loss. Figure 2 shows the innovation variance of the multiple local filters. The result indicates that the measurements of the multi-sensor system can still maintain stable estimates when the measurement data is lost. In Figure 3, Figure 4 and Figure 5, the state estimation, state error, and RMSE of the state are shown for the communication rate $p_{k}^{1}=0.4$, $p_{k}^{2}=0.7$ for the proposed algorithm and the EKF algorithm in the framework of this paper, respectively. The results show that the state estimation results of the proposed algorithm are more accurate and stable compared to the EKF. Figure 6 and Figure 7 record the state estimation results for local estimation and sequential estimation. The figures show that the multi-sensor estimation for the sequential fusion is more accurate than the partial estimation, which implies that the proposed algorithm is more reliable when the system generates data packet dropout.

Table 1 shows the results of the partial and full fusion of the proposed algorithm and the RMSE of the EKF at different communication rates when the packet dropout is slight. The simulation results show that the estimation accuracy of the proposed algorithm is significantly better than that of the EKF under normal packet dropout, with a 58.95% improvement in relative accuracy. Again, it is demonstrated that all fusion has better accuracy compared to partial sensor fusion. Table 2 shows a comparison of the results of the root mean square error of the two algorithms when all sensors suffer from severe packet dropout (all sensors have a communication rate below 50%), resulting in a very low communication rate. We can analyze the state estimation of the EKF scatters in this case, especially when the sensor communication rate is $p_{k}^{1}=0.35,p_{k}^{2}=0.45$. The proposed algorithm is able to maintain a stable accuracy under such extreme conditions, demonstrating that the proposed algorithm can maintain strong robustness in multi-sensor nonlinear systems.

## 6. Conclusions

In this paper, a sequential fusion filtering algorithm is proposed for a nonlinear multi-sensor system with cross-correlated noise and packet dropout compensation, based on innovation analysis and the measurement prediction compensation mechanism. In the case of synchronous correlation of observation noise of different sensors at the same time, the observation noise of sensors at the current moment is correlated with the process noise of the previous moment and packet dropout of measurement data, the measurement data is updated in real time by combining the measurement prediction compensation mechanism and the observation noise estimation to avoid the noise de-correlation process. A sequential fusion filter is designed according to the innovation analysis method, and finally, a numerical implementation step of the sequential fusion filter is given based on the third-degree spherical-radial cubature rule. Simulation results verify the effectiveness of the sequential fusion filter algorithm proposed in this paper.

## Figures and Tables

**Table 1:** RMSE and communicate rates for CKF and EKF.

$p_{k}^{i}$	$p_{k}^{1}=0.35$, $p_{k}^{2}=0.75$	$p_{k}^{1}=0.4$, $p_{k}^{2}=0.7$	$p_{k}^{1}=0.45$, $p_{k}^{2}=0.8$	$p_{k}^{1}=0.45$, $p_{k}^{2}=0.85$
RMSE of CKF for partial sensor	5.201001	4.479368	5.000045	5.231011
RMSE of CKF	4.680037	3.822499	3.844220	4.195554
RMSE of EKF	10.094846	10.010046	10.017550	10.166850

**Table 2:** RMSE and low communicate rates for CKF and EKF.

$p_{k}^{i}$	$p_{k}^{1}=0.35,p_{k}^{2}=0.45$	$p_{k}^{1}=0.37,p_{k}^{2}=0.40$	$p_{k}^{1}=0.4,p_{k}^{2}=0.3$
RMSE of CKF for partial sensor	6.387763	6.003190	7.399094
RMSE of CKF	5.967350	5.470605	7.035220
RMSE of EKF	100.083639	28.344192	40.162560

## Appendix A

The nonlinear multi-sensor sequential fusion state estimation algorithm with cross-correlated noise and packet dropout compensation is proved as follows.

### Appendix A.1. System Sequential Fusion State Estimation with Correlated Noise and Packet Dropout Compensation

The proofs of the state estimate of the *i*th sensor ${\hat{X}}_{k+1}^{i}$, the state estimation error covariance matrix $P_{k+1\left|k+1\right.}^{X^{i}}$, the filter gain $M_{k+1}^{X^{i}}$, the innovation $\varepsilon_{i}\left(k+1\right)$, and the innovation covariance matrix $Q_{\varepsilon }^{i}\left(k+1\right)$ are first given below.

According to the projection theorem, at the time step k+1, the state estimate of the *i*th sensor ${\hat{X}}_{k+1}^{i}$ as:

(A1) $$\begin{array}{cc} {\hat{X}}_{k+1}^{i} & =E\left[X_{k+1}|\Pi \left(k\right),\Pi_{i}\left(k+1\right)\right]\\ \; & =E\left[X_{k+1}|\Pi \left(k\right),\Pi_{i-1}\left(k+1\right),Y_{k+1}^{\left(i\right)}\right]\\ \; & =E\left[X_{k+1}|\Pi \left(k\right),\Pi_{i-1}\left(k+1\right)\right]+M_{k+1}^{X^{i}}\varepsilon_{i}\left(k+1\right)\\ \; & ={\hat{X}}_{k+1}^{i-1}+M_{k+1}^{X^{i}}\varepsilon_{i}\left(k+1\right) \end{array}$$

Then, (7) is obtained.

The filter gain expression $M_{k+1}^{X^{i}}$ is derived as follows:

(A2) $$\begin{array}{cc} M_{k+1}^{X^{\prime }} & =P_{k+1|k+1}^{X^{t-1}Y^{k+1}}{\left(Q_{\varepsilon }^{i}\left(k+1\right)\right)}^{-1}\\ \; & =E\left[\left(X_{k+1}-E\left[X_{k+1}|\Pi \left(k\right),\Pi_{i-1}\left(k+1\right)\right]\right)\right.\\ \; & \left.\left(Y_{k+1}^{\left(i\right)}-E\left[Y_{k+1}^{\left(i\right)}|\Pi \left(k\right),\Pi_{i-1}\left(k+1\right)\right]\right)\right]\\ \; & \times \left(E\left[\left(Y_{k+1}^{\left(i\right)}-E\left[Y_{k+1}^{\left(i\right)}|\Pi \left(k\right),\Pi_{i-1}\left(k+1\right)\right]\right)\right.\right.\\ \; & {\left.\left.\left(Y_{k+1}^{\left(i\right)}-E\left[Y_{k+1}^{\left(i\right)}|\Pi \left(k\right),\Pi_{i-1}\left(k+1\right)\right]\right)\right]\right)}^{-1} \end{array}$$

The system state estimation error ${\tilde{X}}_{k+1}^{i}$ is obtained by subtracting ${\hat{X}}_{k+1}^{i}$ from $X_{k+1}$:

(A3) $$\begin{array}{cc} {\tilde{X}}_{k+1}^{i} & =X_{k+1}-{\hat{X}}_{k+1}^{i}\\ \; & =X_{k+1}-{\hat{X}}_{k+1}^{i-1}-M_{k+1}^{X^{i}}\varepsilon_{i}\left(k+1\right)\\ \; & ={\tilde{X}}_{k+1}^{i-1}-M_{k+1}^{X^{i}}\varepsilon_{i}\left(k+1\right) \end{array}$$

The system state estimation error covariance of the *i*th sensor $P_{k+1\left|k+1\right.}^{X^{i}}$ is:

(A4) $$P_{k+1\left|k+1\right.}^{X^{i}}=E\left[{\tilde{X}}_{k+1}^{i}{\left(◾\right)}^{T}\left|\Pi \left(k\right),\Pi_{i-1}\left(k+1\right)\right.\right]$$

Then, substituting (A3) into (A4) leads to (8). Moreover, (7) and (8) in this paper are proven.

From the definition of the innovation, at the time step k+1 the innovation $\varepsilon_{i}\left(k+1\right)$ of the *i*th sensor is:

(A5) $$\begin{array}{cc} \varepsilon_{i}\left(k+1\right) & =Y_{k+1}^{\left(i\right)}-Y_{k+1}^{\left(i|i-1\right)}\\ \; & =Y_{k+1}^{\left(i\right)}-E\left[Y_{k+1}^{\left(i\right)}|\Pi \left(k\right),\Pi_{i-1}\left(k+1\right)\right] \end{array}$$

Then, substituting (3) into (A5) leads to (9).

The proof of the innovation covariance matrix $Q_{\varepsilon }^{i}\left(k+1\right)$ are given as follows:

(A6) $$\begin{array}{cc} Q_{\varepsilon }^{i}\left(k+1\right) & =E\left[\varepsilon_{i}\left(k+1\right){\left(\varepsilon_{i}\left(k+1\right)\right)}^{T}|\Pi \left(k\right),\Pi_{i-1}\left(k+1\right)\right]\\ \; & =E\left[\left(Y_{k+1}^{\left(i\right)}-E\left[Y_{k+1}^{\left(i\right)}|\Pi \left(k\right),\Pi_{i-1}\left(k+1\right)\right]\right){\left(•\right)}^{T}|\Pi \left(k\right),\Pi_{i-1}\left(k+1\right)\right] \end{array}$$

substituting (3) into (A6) leads to (10), where

(A7) $$\begin{array}{cc} \; & E\left[\left(Z_{k+1}^{\left(i\right)}\right){\left(\cdot \right)}^{T}|\Pi \left(k\right),\Pi_{i-1}\left(k+1\right)\right]\\ & =\left[\left[\left(H^{\left(i\right)}\left(X_{k+1}\right)+V_{k+1}^{\left(i\right)}\right){\left(H^{\left(i\right)}\left(X_{k+1}\right)+V_{k+1}^{\left(i\right)}\right)}^{T}|\Pi \left(k\right),\Pi_{i-1}\left(k+1\right)\right]\right.\\ \; & =E\left[H^{\left(i\right)}\left(X_{k+1}\right){\left(H^{\left(i\right)}\left(X_{k+1}\right)\right)}^{T}|\Pi \left(k\right),\Pi_{i-1}\left(k+1\right)\right]\\ \; & +E\left[H^{\left(i\right)}\left(X_{k+1}\right){\left(V_{k+1}^{\left(i\right)}\right)}^{T}|\Pi \left(k\right),\Pi_{i-1}\left(k+1\right)\right]\\ \; & +E\left[V_{k+1}^{\left(i\right)}{\left(H^{\left(i\right)}\left(X_{k+1}\right)\right)}^{T}|\Pi \left(k\right),\Pi_{i-1}\left(k+1\right)\right]+E\left[V_{k+1}^{\left(i\right)}{\left(V_{k+1}^{\left(i\right)}\right)}^{T}|\Pi \left(k\right),\Pi_{i-1}\left(k+1\right)\right]\\ \; & =\int H^{\left(i\right)}\left(X_{k+1}\right){\left(H^{\left(i\right)}\left(X_{k+1}\right)\right)}^{T}N\left(X_{k+1};{\hat{X}}_{k+1}^{i-1},P_{k+1+k+1}^{X^{t-1}}\right)dX_{k+1}\\ \; & +\left(\int H^{\left(i\right)}\left(X_{k+1}{\left(V_{k+1}^{\left(i\right)}\right)}^{T}N\left(\left[\begin{array}{c} X_{k+1}\\ V_{k+1}^{\left(i\right)} \end{array}\right];\left[\begin{array}{c} {\hat{X}}_{k+1}^{i-1}\\ {\hat{V}}_{k+1}^{\left(i-1\right)} \end{array}\right],\Psi_{1}\right)d\left[\begin{array}{c} X_{k+1}\\ V_{k+1}^{\left(i\right)} \end{array}\right]\right)+{\left(\cdot \right)}^{T}+R_{k+1}^{i}\right. \end{array}$$

(A8) $$\begin{array}{cc} Z_{k+1|k}^{\left(i\right)} & =E\left[Z_{k+1}^{\left(i\right)}|\Pi \left(k\right)\right]=E\left[H^{\left(i\right)}\left(X_{k+1}\right)+V_{k+1}^{\left(i\right)}|\Pi \left(k\right)\right]\\ \; & =\int H^{\left(i\right)}\left(X_{k+1}\right)N\left(X_{k+1};{\hat{X}}_{k+1|k}^{0},P_{k+1|k}^{X^{0}}\right)dX_{k+1} \end{array}$$

(A9) $$\begin{array}{cc} Z_{k+1|k+1}^{\left(i|i-1\right)} & =E\left[Z_{k+1}^{\left(i\right)}|\Pi \left(k\right),\Pi_{i-1}\left(k+1\right)\right]\\ \; & =E\left[H^{\left(i\right)}\left(X_{k+1}\right)+V_{k+1}^{\left(i\right)}|\Pi \left(k\right),\Pi_{i-1}\left(k+1\right)\right]\\ \; & =\int H^{\left(i\right)}\left(X_{k+1}\right)N\left(X_{k+1};{\hat{X}}_{k+1}^{i-1},P_{k+1|k+1}^{X^{i-1}}\right)dX_{k+1}+V_{k+1|k+1}^{\left(i|i-1\right)} \end{array}$$

The covariance matrix between the state estimation error and the innovation is:

(A10) $$\begin{array}{cc} P_{k+1|k+1}^{X^{i-1}Y^{i|i-1}} & =E\left[\left(X_{k+1}-{\hat{X}}_{k+1}^{i-1}\right){\left(Y_{k+1}^{\left(i\right)}-Y_{k+1}^{\left(i|i-1\right)}\right)}^{T}|\Pi \left(k\right),\Pi_{i-1}\left(k+1\right)\right]\\ \; & =E\left[\left(X_{k+1}-{\hat{X}}_{k+1}^{i-1}\right)\varepsilon_{i}{\left(k+1\right)}^{T}|\Pi \left(k\right),\Pi_{i-1}\left(k+1\right)\right]\\ \; & =E\left[X_{k+1}{\left(Y_{k+1}^{\left(i\right)}\right)}^{T}|\Pi \left(k\right),\Pi_{i-1}\left(k+1\right)\right]-{\hat{X}}_{k+1}^{i-1}{\left(Y_{k+1}^{\left(i|i-1\right)}\right)}^{T} \end{array}$$

Then, substituting (3) and (9) into (A10) leads to (12).

### Appendix A.2. Observed Noise Estimation Based on Sequential Fusion with Cross-Correlated Noise and Packet Dropout Compensation

We give the estimates of the observed noise $V_{k+1\left|k+1\right.}^{\left(i\left|i-1\right.\right)}$, the gain matrix $M_{k+1}^{V^{i\left|i-1\right.}}$ and the respective covariance matrices of the system in this paper, and its iterative framework.

According to the projection theorem, the estimation of the observation noise $V_{k+1\left|k+1\right.}^{\left(i\left|i-1\right.\right)}$ as:

(A11) $$\begin{array}{cc} V_{k+1|k+1}^{\left(i|i-1\right)} & =E\left[V_{k+1}^{\left(i\right)}|\Pi \left(k\right),\Pi_{i-1}\left(k+1\right)\right]\\ \; & =E\left[V_{k+1}^{\left(i\right)}|\Pi \left(k\right),\Pi_{i-2}\left(k+1\right),Y_{k+1}^{\left(i-1\right)}\right]\\ \; & =E\left[V_{k+1}^{\left(i\right)}|\Pi \left(k\right),\Pi_{i-2}\left(k+1\right)\right]+M_{k+1}^{V^{i-1}}\varepsilon_{i-1}\left(k+1\right)\\ \; & =V_{k+1|k+1}^{\left(i|i-2\right)}+M_{k+1}^{V^{i-1}}\varepsilon_{i-1}\left(k+1\right) \end{array}$$

(A12) $$V_{k+1\left|k+1\right.}^{\left(i\left|0\right.\right)}=E\left[V_{k+1}^{\left(i\right)}\left|\Pi (k)\right.\right]=0$$

The (A11) can be iterated to obtain (17), where the observation noise gain matrix $M_{k+1}^{V^{i\left|l\right.}}$ is:

(A13) $$\begin{array}{cc} M_{k+1}^{V^{\left(i|l\right)}} & =E\left[V_{k+1}^{\left(i\right)}{\left(\varepsilon_{l}\left(k+1\right)\right)}^{T}|\Pi \left(k\right),\Pi_{l-1}\left(k+1\right)\right]{\left(Q_{\varepsilon }^{l}\left(k+1\right)\right)}^{-1}\\ \; & =E\left[V_{k+1}^{\left(i\right)}{\left(\gamma_{k+1}^{l}Z_{k+1}^{\left(l\right)}+\left(p_{k+1}^{l}-\gamma_{k+1}^{l}\right)Z_{k+1|k}^{\left(l\right)}-p_{k+1}^{l}Z_{k+1|k+1}^{\left(l|l-1\right)}\right)}^{T}|\Pi \left(k\right),\Pi_{l-1}\left(k+1\right)\right]\\ \; & *{\left(Q_{\varepsilon }^{l}\left(k+1\right)\right)}^{-1}\\ \; & =p_{k+1}^{l}\left(\begin{array}{c} E\left[V_{k+1}^{\left(i\right)}{\left(Z_{k+1}^{\left(l\right)}\right)}^{T}|\Pi \left(k\right),\Pi_{l-1}\left(k+1\right)\right]\\ -E\left[V_{k+1}^{\left(i\right)}{\left(Z_{k+1|k+1}^{\left(l|l-1\right)}\right)}^{T}|\Pi \left(k\right),\Pi_{l-1}\left(k+1\right)\right] \end{array}\right]{\left(Q_{\varepsilon }^{l}\left(k+1\right)\right)}^{-1} \end{array}$$

The covariance matrix $P_{k+1\left|k+1\right.}^{V^{i\left|l-1\right.}}$ of the estimation error of the observation noise is:

(A14) $$\begin{array}{cc} P_{k+1|k+1}^{v^{i|l-1}} & =E\left[{\tilde{V}}_{k+1}^{\left(i|l-1\right)}{\left({\tilde{V}}_{k+1}^{\left(i|l-1\right)}\right)}^{T}|\Pi \left(k\right),\Pi_{l-1}\left(k+1\right)\right]\\ \; & =E\left[\left({\tilde{V}}_{k+1}^{\left(i|l-1\right)}-M_{k+1}^{V^{i|l}}\varepsilon_{l}\left(k+1\right)\right){\left({\tilde{V}}_{k+1}^{\left(i|l-1\right)}-M_{k+1}^{v^{i|l}}\varepsilon_{l}\left(k+1\right)\right)}^{T}|\Pi \left(k\right),\Pi_{l-1}\left(k+1\right)\right]\\ \; & =P_{k+1|k+1}^{V^{i|l-1}}-M_{k+1}^{V^{i|l}}Q_{\varepsilon }^{l}\left(k+1\right){\left(M_{k+1}^{V^{i|l}}\right)}^{T} \end{array}$$

(A15) $$\begin{array}{cc} P_{k+1|k+1}^{V^{i|0}} & =E\left[\left(V_{k+1}^{\left(i\right)}-{\hat{V}}_{k+1}^{\left(i|0\right)}\right){\left(V_{k+1}^{\left(i\right)}-{\hat{V}}_{k+1}^{\left(i|0\right)}\right)}^{T}|\Pi \left(k\right)\right]\\ \; & =E\left[V_{k+1}^{\left(i\right)}{\left(V_{k+1}^{\left(i\right)}\right)}^{T}|\Pi \left(k\right)\right]\\ \; & =R_{i}\left(k+1\right) \end{array}$$

(A14) can be iterated to obtain (24).

The covariance matrix $P_{k+1\left|k+1\right.}^{X^{l-1}V^{i\left|l-1\right.}}$ between the state estimation error and the observation noise can be calculated as follows:

(A16) $$\begin{array}{cc} \; & P_{k+1|k+1}^{X^{l-1}V^{i|l-1}}=P_{k+1|k+1}^{X^{l-1}V^{l-1}}=E\left[{\tilde{X}}_{k+1}^{l-1}{\left({\tilde{V}}_{k+1}^{\left(i|l-1\right)}\right)}^{T}|\Pi_{l-1}\left(k+1\right)\right]\\ \; & =E\left[\left({\tilde{X}}_{k+1}^{l-2}-M_{k+1}^{X^{l-1}}\varepsilon_{l-1}\left(k+1\right)\right)\times {\left({\tilde{V}}_{k+1}^{i|l-2}-M_{k+1}^{V^{i|l-1}}\varepsilon_{l-1}\left(k+1\right)\right)}^{T}|\Pi_{l-2}\left(k+1\right)\right]\\ \; & +E\left[\left({\tilde{X}}_{k+1}^{l-2}-M_{k+1}^{X^{l-1}}\varepsilon_{l-1}\left(k+1\right)\right){\left(M_{k+1}^{V^{i|l-1}}\varepsilon_{l-1}\left(k+1\right)\right)}^{T}|\Pi_{l-2}\left(k+1\right)\right]\\ \; & +E\left[\left(M_{k+1}^{X^{l-1}}\varepsilon_{l-1}\left(k+1\right)\right){\left({\tilde{X}}_{k+1}^{l-2}-M_{k+1}^{V^{i|l-1}}\varepsilon_{l-1}\left(k+1\right)\right)}^{T}|\Pi_{l-2}\left(k+1\right)\right]\\ \; & +E\left[\left(M_{k+1}^{X^{l-1}}\varepsilon_{l-1}\left(k+1\right)\right){\left(M_{k+1}^{V^{i|l-1}}\varepsilon_{l-1}\left(k+1\right)\right)}^{T}|\Pi_{l-2}\left(k+1\right)\right]\\ \; & =P_{k+1|k+1}^{X^{l-2}V^{i|l-2}}-K_{k+1}^{X^{l-1}}Q_{\varepsilon }^{l-1}\left(k+1\right){\left(M_{k+1}^{V^{i|l-1}}\right)}^{T} \end{array}$$

(A17) $$\begin{array}{cc} {\hat{X}}_{k+1}^{0} & =E\left[X_{k+1}|\Pi \left(k\right)\right]=E\left[F\left(X_{k}\right)+\Gamma_{k}W_{k}|\Pi \left(k\right)\right]\\ \; & =\int F\left(X_{k}\right)N\left(X_{k};{\hat{X}}_{k|k}^{L},P_{k|k}^{X^{L}}\right)dX_{k} \end{array}$$

(A18) $$\begin{array}{cc} P_{k+1|k+1}^{X^{0}V^{i|0}} & =E\left[\left(X_{k+1}-{\hat{X}}_{k+1}^{0}\right){\left(V_{k+1}^{\left(i\right)}-{\hat{V}}_{k+1}^{\left(i|0\right)}\right)}^{T}|\Pi \left(k\right)\right]\\ \; & =E\left[\left(\begin{array}{c} F\left(X_{k}\right)+\Gamma_{k}W_{k}\\ -\int F\left(X_{k}\right)N\left(X_{k};{\hat{X}}_{k|k}^{N},P_{k|k}^{X^{N}}\right)dX_{k} \end{array}\right){\left(V_{k+1}^{\left(i\right)}\right)}^{T}|\Pi \left(k\right)\right]\\ \; & =\int F\left(X_{k}\right)N\left(X_{k};{\hat{X}}_{k|k}^{N},P_{k|k}^{X^{N}}\right)dX_{k}{\left({\hat{V}}_{k+1}^{\left(i|0\right)}\right)}^{T}\\ \; & -\int F\left(X_{k}\right)N\left(X_{k};{\hat{X}}_{k|k}^{N},P_{k|k}^{X^{N}}\right)dX_{k}{\left({\hat{V}}_{k+1}^{\left(i|0\right)}\right)}^{T}+\Gamma_{k}E\left[W_{k}{\left(V_{k+1}^{\left(i\right)}\right)}^{T}\right]\\ \; & =S_{i}\left(k\right) \end{array}$$

(A16) can be iterated to obtain (23).

Thus, Equation (21) completes the proof by the above description.
